# Koala cathelicidin PhciCath5 has antimicrobial activity, including against *Chlamydia pecorum*

**DOI:** 10.1371/journal.pone.0249658

**Published:** 2021-04-14

**Authors:** Emma Peel, Yuanyuan Cheng, Julianne T. Djordjevic, Denis O’Meally, Mark Thomas, Michael Kuhn, Tania C. Sorrell, Wilhelmina M. Huston, Katherine Belov

**Affiliations:** 1 School of Life and Environmental Sciences, The University of Sydney, Sydney, New South Wales, Australia; 2 Centre for Infectious Diseases and Microbiology, The Westmead Institute for Medical Research, Westmead, New South Wales, Australia; 3 Marie Bashir Institute for Infectious Diseases and Biosecurity, The University of Sydney, Westmead, New South Wales, Australia; 4 Center for Gene Therapy, Beckman Research Institute of the City of Hope, Duarte, California, United States of America; 5 School of Life Sciences, University of Technology Sydney, Sydney, New South Wales, Australia; 6 Zoetis, Veterinary Medicine Research and Development, Kalamazoo, Michigan, United States of America; Midwestern University, UNITED STATES

## Abstract

Devastating fires in Australia over 2019–20 decimated native fauna and flora, including koalas. The resulting population bottleneck, combined with significant loss of habitat, increases the vulnerability of remaining koala populations to threats which include disease. *Chlamydia* is one disease which causes significant morbidity and mortality in koalas. The predominant pathogenic species, *Chlamydia pecorum*, causes severe ocular, urogenital and reproductive tract disease. In marsupials, including the koala, gene expansions of an antimicrobial peptide family known as cathelicidins have enabled protection of immunologically naïve pouch young during early development. We propose that koala cathelicidins are active against *Chlamydia* and other bacteria and fungi. Here we describe ten koala cathelicidins, five of which contained full length coding sequences that were widely expressed in tissues throughout the body. Focusing on these five, we investigate their antimicrobial activity against two koala *C*. *pecorum* isolates from distinct serovars; MarsBar and IPTaLE, as well as other bacteria and fungi. One cathelicidin, PhciCath5, inactivated *C*. *pecorum* IPTaLE and MarsBar elementary bodies and significantly reduced the number of inclusions compared to the control (p<0.0001). Despite evidence of cathelicidin expression within tissues known to be infected by *Chlamydia*, natural PhciCath5 concentrations may be inadequate *in vivo* to prevent or control *C*. *pecorum* infections in koalas. PhciCath5 also displayed antimicrobial activity against fungi and Gram negative and positive bacteria, including methicillin-resistant *Staphylococcus aureus* (MRSA). Electrostatic interactions likely drive PhciCath5 adherence to the pathogen cell membrane, followed by membrane permeabilisation leading to cell death. Activity against *E*. *coli* was reduced in the presence of 10% serum and 20% whole blood. Future modification of the PhciCath5 peptide to enhance activity, including in the presence of serum/blood, may provide a novel solution to *Chlamydia* infection in koalas and other species.

## Introduction

The koala (*Phascolarctos cinereus*) is an iconic Australian marsupial and the last surviving member of the Phascolarctidae. Marsupials are one of three mammalian lineages, the others being eutherian mammals such as humans, and monotremes such as the platypus. Marsupials differ from other mammals in a number of key anatomical and physiological traits, many of which are involved in reproduction and development [1]. Koalas are mostly arboreal marsupials that subsist on a strict diet of *Eucalyptus* leaves [1]. Typical of marsupials, koalas have a short gestation period of up to 35 days and give birth to altricial young that remain in the pouch for 9 months [1].

Fires devastated large swathes of Australia in 2019–20, burning through at least 11 million hectares (1.1x10^11^ m^2^), destroying crucial habitat of already vulnerable and threatened species, and driving many to the brink of extinction [2,3]. Estimates suggest nearly three billion animals were killed or impacted by the fires [4]. In response the Australian Government identified 119 priority species that require urgent management intervention, one of which was the koala [3]. Prior to this catastrophic event, koala populations were already in decline along the east coast of Australia due to multiple threats including habitat loss, climate change, and disease [5,6]. The 2019–20 fires further decimated these populations; with at least 3.5 million hectares(3.5 x 10^10^ m^2^), or 25%, of koala suitable habitat in eastern NSW affected by fire [7]. The resulting genetic bottleneck, combined with substantial habitat destruction by fire, leaves remaining populations especially vulnerable to new and existing threats including disease [5,8].

Three main diseases infect koalas; koala retrovirus [9], the fungus, *Cryptococcus* [10], and the bacterium *Chlamydia* [5]. Chlamydiosis, the disease resulting from *Chlamydia* infection, is a major contributing factor to the decline and long-term viability of koala populations [5]. *Chlamydia* are intracellular, Gram-negative bacteria which infect a wide range of hosts including humans, livestock, and wildlife [11]. They have a bi-phasic life cycle consisting of an intracellular reticulate body that is metabolically active and replicates within the host cell, and an infectious elementary body (EB) that is released into the extracellular environment following host cell rupture. *Chlamydia pecorum* is principally responsible for chlamydiosis in koalas, and causes both mild and severe disease [6,12]. Clinical manifestations include ocular disease leading to blindness, urogenital disease resulting in cystitis and infertility, and respiratory disease [5]. The prevalence of infection varies, but is as high as 90% in koala populations in Queensland, New South Wales, and Victoria [5,6].

Substantial research over the past decade has culminated in a promising *C*. *pecorum* vaccine for koalas (reviewed in [13]). However, limitations remain regarding long-term protection against reinfection [14], hence research is ongoing and treatment remains an essential component of the response to chlamydiosis in koalas. Treating chlamydiosis in koalas can be difficult as macrolide and tetracycline antibiotics commonly used in humans cause gastrointestinal dysbiosis, which can be fatal [15,16]. Chloramphenicol and enrofloxacin are commonly used, and pharmacokinetic studies have aided in developing koala-specific dosage regimes [17–19]. However, koalas continue to shed the pathogen after treatment with enrofloxacin [20]. Chloramphenicol is the mainstay of current treatment regimens, although adverse side-effects have been observed [19,21]. Use of chloramphenicol is further confounded by its decreasing availability [6], driving the search for alternative antibiotics.

Florfenicol, a derivative of chloramphenicol, has yielded mixed results as the highest tolerated dose produced suboptimal plasma concentrations, and the majority of infections required additional treatments or did not resolve [22]. Doxycycline effectively cleared the infection, but only a single study of five koalas has been conducted [23]. Natural innate defence mechanisms of the koala, including antimicrobial peptides (AMPs), may play a role in reducing chlamydial infection and provide avenues for new treatment options in the future.

There are two main families of AMPs in mammals; cathelicidins and defensins [24]. Cathelicidins are small, cationic antimicrobial peptides expressed within neutrophils and epithelial cells, and are features of the innate immune system [24]. They have both immunomodulatory and antimicrobial functions, and display activity against a range of bacteria, fungi and viruses [24]. Throughout evolution cathelicidins have expanded in marsupials, compared to eutherian mammals, resulting in numerous diverse peptides [25–28]. For example, the gray short-tailed opossum has 19 cathelicidin genes [27,28], while humans have only one [29]. Expansions within marsupials are likely driven by the need to protect immunologically naive young during pouch life [25,30]. Marsupials have a very short gestation period of up to 35 days and give birth to altricial young which are immunologically naïve at birth [1,31]. During immunological development the young encounter a diverse range of microbial flora within the pouch [32], and are protected by products of innate immune mechanisms such as cathelicidins expressed in the milk [30,33] and pouch lining [25,34]. Previous work has shown that tammar wallaby and Tasmanian devil cathelicidins have potent broad spectrum antimicrobial activity and kill drug resistant bacteria such as methicillin-resistant *S*. *aureus* (MRSA) [30] and multidrug-resistant isolates of *Klebsiella pneumoniae*, *Pseudomonas aeruginosa and Acinetobacter baumannii* [35]. However, activity against intracellular bacteria such as *Chlamydia* has not been tested. Cathelicidins from humans and livestock inactivated a number of *Chlamydia* species but were ineffective against *C*. *pecorum* [36–39].

Our aim was to characterise cathelicidins in the koala genome [40] and transcriptomes [41,42], and determine the activity of five synthetic cathelicidins against two koala *C*. *pecorum* strains; IPTaLE and MarsBar, as well as other bacteria and fungi from humans and animals. To further understand the mechanism of antimicrobial activity, we assessed membrane permeabilisation and activity in the presence of inhibitors. The distribution of cathelicidin transcripts across a range of tissues was examined to determine if cathelicidins are present at the site of *Chlamydia* infection, and hence may be involved in natural defence against *Chlamydia*.

## Methods

### Bioinformatics

Koala cathelicidins were identified in the koala genome [40] and transcriptome [41,42] using BLAST with default parameters, and previously characterised marsupial, monotreme and eutherian cathelicidins as query sequences (S3 Table). Multiple sequence alignments of putative koala cathelicidins with sequences from other marsupial, monotreme and eutherian cathelicidins (S3 Table) were constructed using ClustalW [43] in BioEdit [44] to identify conserved peptide domains and motifs. Signal peptide sequences were predicted using SignalP 4.1 [45]. To examine phylogenetic relationships, amino acid alignments of full-length sequences, and cathelin domain only, were used to construct individual phylogenetic trees in MEGA7 [46] using the neighbour-joining method with p-distance, pairwise deletion and 500 bootstrap replicates, as well as maximum likelihood method, with the Jones Taylor-Thornton model and 500 bootstrap replicates. Both neighbour-joining and maximum likelihood methods produced the same tree topology for alignments of full-length sequences and cathelin domain only, hence only the maximum likelihood trees are displayed here.

Only full-length sequences with complete open reading frames were included in subsequent analyses. The relative transcription levels of full-length koala cathelicidins were examined in previously published liver, spleen, bone marrow, lymph, lung, kidney, testis, uterus, brain, salivary gland, adrenal gland, and mammary gland transcriptomes from one koala euthanized due to unsuccessful treatment for severe chlamydiosis and one koala euthanized due to dog attack [41,42]. RNAseq reads (SRR1106690, SRR1106707, SRR1121764, SRR1122141, SRR1203868, SRR1205138, SRR1205176, SRR1205218, SRR1205222-SRR1205224, SRR1205998, SRR1207974, SRR1207975, SRR3724381) were mapped against the koala assembly (GCF_002099425.1) using STAR [47] and abundance estimated using Stringtie [48] as transcripts per million (TPM).

Mature peptide cleavage sites were predicted using ExPasy peptide cutter (http://web.expasy.org/peptide_cutter/) with neutrophil elastase. Molecular weight of mature peptides and charge at pH 7 was calculated using Protein Calculator v3.4 (http://protcalc.sourceforge.net/, May 2013). Hydrophobicity percentage was calculated using Peptide 2.0 hydrophobicity/hydrophilicity analysis (http://peptide2.cpm/N_peptide_hydrophobicity_hydrophilicity.php, 2016). Kyte and Dolittle hydropathicity plots [49] and Deleage and Roux alpha helicity plots [50], both with a window size of n = 7, were created using ProtScale through the ExPasy server [51]. Grand average of hydropathicity (GRAVY) scores were calculated using ProtParam through the ExPasy server [51]. Mature peptide amino acid similarity scores were calculated in BioEdit [44] using the BLOSUM62 matrix. Mature peptides were synthesised by ChinaPeptides Co. Ltd. to >95% purity.

### Antimicrobial susceptibility

Antimicrobial activity was determined against a range of bacteria and fungi from humans and animals using a broth microdilution susceptibility assay according to clinical laboratory standards institute (CLSI) guidelines in 96 well polypropylene plates as described previously [30]. Bacterial and fungal isolates tested are summarised in Table 2. Briefly, cathelicidins were dissolved in DMSO and serially diluted, in cation-adjusted Mueller Hinton Broth (MH II B) with or without 10% lysed horse blood for bacteria, and yeast nitrogen base (YNB) for fungi. Cathelicidin concentrations ranged between 64μg/mL and 0.125μg/mL in a final volume of 100μL. For all bacteria and fungi tested, ampicillin, tetracycline and fluconazole were included as positive controls, in addition to a media-only control and growth control (no inhibitor). Bacteria and fungi were sub-cultured 20–24 hours prior to the test, suspended in saline and their concentration adjusted to a 0.5 McFarland standard. Microorganisms were then diluted to a concentration of 0.5–1.0x10^6^ cells/mL, with colony counts performed to confirm microorganism density, and 100μL was dispensed into the wells of the cathelicidin dilution plate. All plates were incubated at 35°C for 20–48 hours depending on the strain. Antimicrobial activity was expressed as minimum inhibitory concentration (MIC), which was defined as the lowest concentration of cathelicidin preventing visible bacterial growth, relative to the no-drug control. The same microdilution susceptibility assay was performed using Mueller Hinton Broth without the addition of the divalent cations calcium and magnesium (MHB), to test the effect of the cations on PhciCath5 activity against the ATCC strains *E*. *coli* 25922 and *S*. *aureus* 29213.

### Effect of serum and blood on antibacterial activity

The potential inhibitory effect of serum and blood on PhciCath5 antibacterial activity was investigated using a broth microdilution susceptibility assay as described above with the following modifications. This assay was performed in collaboration with Zoetis Veterinary Medicine Research and Development. PhciCath5 was solubilized in water for cell culture containing 0.01% acetic acid and serial two-fold dilutions were prepared from 50mM to 0.78mM. *E*. *coli* ATCC25922 was sub-cultured onto sheep blood agar (SAB) and incubated at 35°C for 24 hours prior to the test. Colonies were suspended in saline and the concentration adjusted to a 0.5 McFarland standard. The bacterial suspension was then diluted 1/250 with MHB containing 10% bovine serum albumin (BSA) or 20% whole mouse blood (BioIVT). The cathelicidin serial dilutions were further diluted 1/10 with bacterial suspension in a 96-well polypropylene plate, to give a final cathelicidin concentration ranging between 50 and 0.78μM. A growth control (no inhibitor) was also included. The plates were incubated for 24 hours at 37°C and the MIC recorded as the lowest concentration of cathelicidin preventing visible bacterial growth, relative to the no-drug control.

### Bacterial membrane permeability

Membrane permeabilisation of *E*. *coli* ATCC25922 by PhciCath5 was assessed using the Promega CellTox green cytotoxicity assay. *E*.*coli* ATCC25922 was sub-cultured onto TSA II blood agar and incubated at 35^o^c for 24 hours prior to the test. A bacterial suspension was prepared in RPMI to give an OD_600_ reading of 0.2. PhciCath5 was dissolved in water for cell culture containing 0.01% acetic acid and serial two-fold dilutions prepared in a black 384-well polypropylene plate. The plate was then innoculated with *E*. *coli* ATCC25922, producing a total well volume of 30uL and final peptide concentration of 50 to 0.05uM. Fluorescence was then measured at 512nm using the Perkin Elmer Envision multilabel plate reader (0 hours). The plate was then incubated at room temperature and additional fluorescence measurements were recorded at 1, 2, 3 and 4hrs. Membrane permeability was calculated as a percentage relative to the “no inhibitor” control. PhciCath5 concentration which resulted in greater than or equal to 5% *E*. *coli* ATCC25922 membrane permeability was reported.

### *Chlamydia pecorum* antimicrobial susceptibility

*C*. *pecorum* IPTaLE and MarsBar [11,52] were cultured in mouse McCoy B cells, on DMEM supplemented with 10% foetal calf serum (FCS), 0.1mg/mL streptomycin and 0.05mg/mL gentamicin at 37°C in a 5% CO_2_ atmosphere. Cell lines were routinely tested for mycoplasma contamination every 2 months. Prior to performing antimicrobial susceptibility assays, 96-well plates were seeded with 30,000 host cells per well 24 hours prior to chlamydial infection as described previously [53,54]. For the antimicrobial assays, koala cathelicidin mature peptides, PhciCath1, 2, 3, 5 and 6, were solubilized in water for cell culture with 0.01% acetic acid, and two-fold dilutions were made in sucrose-phosphate-glutamic acid (SPG) media from 1mg to 250μg/mL, in triplicate. Cathelicidin containing wells were diluted one in two with *C*. *pecorum* IPTaLE and MarsBar EBs and incubated for 2 hours at 37°C, giving a final cathelicidin concentration of 500, 250 and 125μg/mL. A negative SPG only control was included. To exclude the possibility of cathelicidin toxicity to McCoy cells, cathelicidin dilutions were removed by centrifugation and *C*. *pecorum* EBs re-suspended in DMEM supplemented with 10% FCS, 0.1mg/mL streptomycin and 0.05mg/mL gentamycin. The suspension was used to infect a McCoy B ATCC CRL-1696 cell monolayer at a Multiplicity of Infection (MOI) of 0.6 as described previously [53,54]. At 44 hours post infection, host cells were lysed by vigorous pipetting and *Chlamydia* harvested by centrifugation. Following one freeze-thaw passage of the supernatant, *Chlamydia* were serially diluted onto fresh McCoy cell monolayers, and fixed and stained at 40hrs post infection for enumeration of *Chlamydia* inclusion forming units (IFU) per mL. This approach involving two rounds of infection essentially provides the minimum chlamydicidal concentration, or the minimum concentration of cathelicidin required to kill *Chlamydia*. Monolayers were stained with DAPI, a polyclonal HtrA antibody and secondary anti-rabbit antibody which stains chlamydial inclusion bodies [52–54], and visualised on the InCell 2200. Statistical analysis was performed on the Prism GraphPad software [55]. A one-way ANOVA followed by a Holm-Sidak’s multiple comparisons test was performed relative to the control.

## Results and discussion

### Characterisation of koala cathelicidins expressed in different tissues

Ten cathelicidins were identified within a 1.3Mb region on scaffold 76 of the koala genome and were named in order of identification (S1 Table). Five cathelicidins, *PhciCath1*, *2*, *3*, *5* and *6*, were full-length and contained complete open reading frames. One cathelicidin, *PhciCath4*, contained a premature stop codon in exon 3 and hence is likely to be a pseudogene. Only partial sequences could be identified for four cathelicidins, *PhciCath7p* through *10p*.

**Fig 1:**
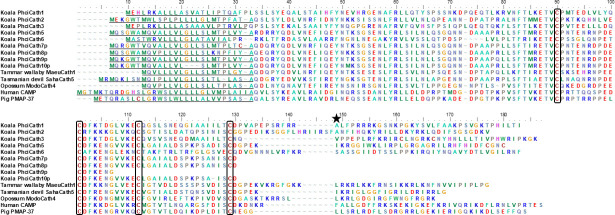
Multiple sequence alignment of koala cathelicidins with other marsupial and eutherian cathelicidins. PhciCath4 was not included in the alignment as it contains a premature stop codon and hence is a likely a pseudogene. PhciCath7p to 10p are partial sequences only, as the mature peptide could not be identified. The predicted signal peptide sequence is underlined, followed by two domains; the cathelin domain which contains conserved cysteine residues (boxed), and the antimicrobial domain which encodes the mature peptide and is of variable length and composition. The predicted mature peptide cleavage site is denoted by a star.

All koala cathelicidins contained sequence features characteristic of the cathelicidin family (Fig 1) [24]. Koala cathelicidin genes contained four exons, which encode a prepropeptide consisting of three domains. The signal peptide and cathelin domain contained conserved stretches of sequence, including four cysteine residues in the latter which are a distinguishing feature of the family and provide structure to the prepropeptide (Fig 1) [24]. For *PhciCath1*, *2*, *3*, *5* and *6* with full-length sequences, the antimicrobial domain which encodes the mature peptide was variable in length and composition (Table 1), with a maximum 30% amino acid similarity amongst the five predicted mature peptide sequences (S1 Table). Tasmanian devil cathelicidins also display a similar level of variability in this domain, however in eutherian mammals such as pigs, amino acid similarity can be as high as 94% [30].

**Table 1:** Physiochemical properties of predicted mature peptides from full-length koala cathelicidins.

Cathelicidin	Sequence	Molecular weight (g/mol)	Charge at pH7	Hydrophobic %	GRAVY score
PhciCath1	LFPRRRKGSNKPGKYSVLFAAKPSVGKTPHILTI	3765.49	8.1	44.12	-0.424
PhciCath2	NFIHQKYRILLDKYRKLQDIFSGSGDKV	3382.90	3.2	32.14	-0.682
PhciCath3	PPEPLRFKRIRCLNGRKCNYHNLLLTIVPHWRIPKGK	4465.38	8.3	43.24	-0.695
PhciCath5	KRGGIWKLIRPLGRGAGRILRHFHIDFCGNC	3548.23	6.3	38.71	-0.219
PhciCath6	ASSGIIDTSSLPPKIRQIYNQAVYDTLVGILRNF	3751.71	0.9	44.12	0.106

All five mature peptides are of a similar molecular weight, and most display a high cationic charge at pH 7. The percentage of hydrophobic amino acids and grand average of hydropathicity (GRAVY) score for each mature peptide is also presented. The GRAVY score measures the hydrophilicity or hydrophobicity of a peptide, with negative and positive scores indicating a hydrophilic and hydrophobic peptide respectively.

**Fig 2:**
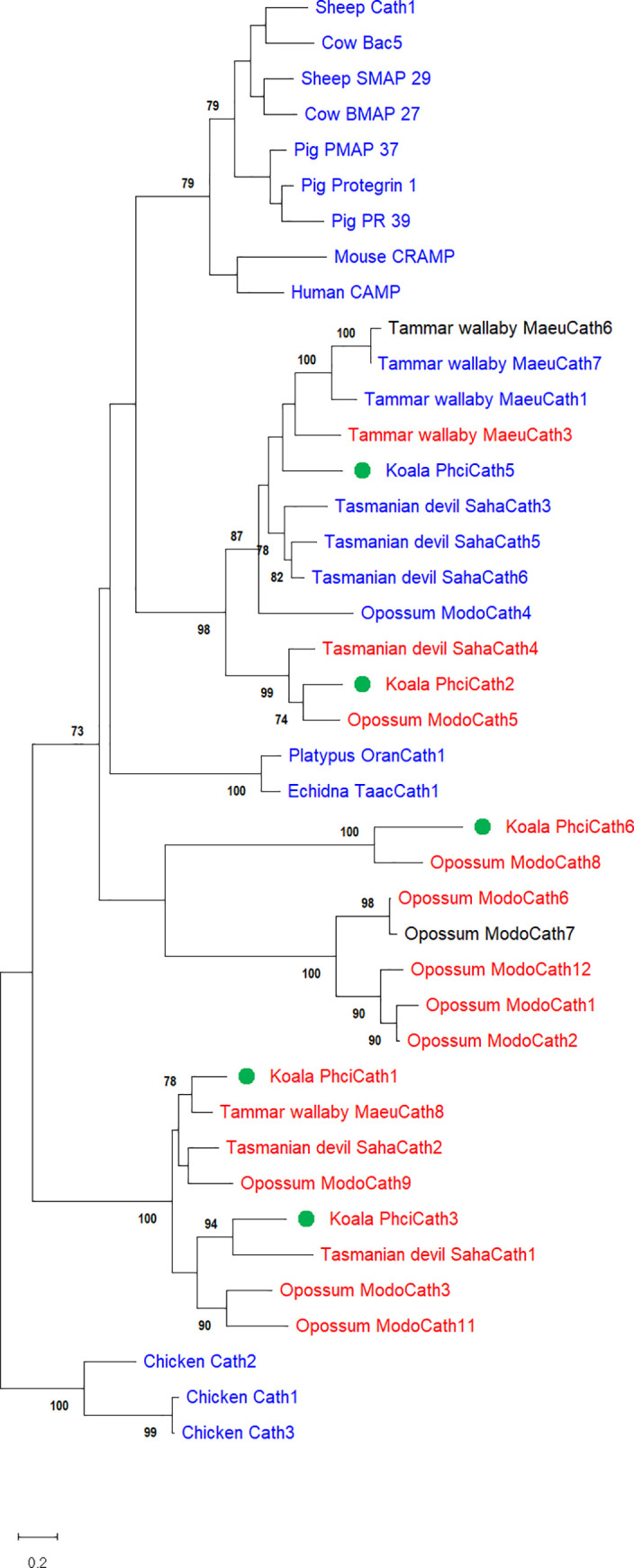
Full-length koala cathelicidins cluster with other marsupials in the phylogenetic tree, particularly PhciCath5 which clusters with other marsupial cathelicidins that display antimicrobial activity. Sequences are coloured according to antimicrobial activity against bacteria and/or fungi; blue indicates active, red indicates inactive and black indicates the peptide has not been tested. Koala cathelicidins are denoted by a green circle. Only bootstrap values greater than 70% are shown. Accession numbers for published sequences used in this tree are available in S3 Table.

Koala cathelicidins cluster with other marsupial cathelicidins in the gene tree, as expected (Fig 2). *PhciCath1*, *3* and *6* are orthologs of *MaeuCath8*, *SahaCath1* and *ModoCath8* respectively, indicating that these genes arose prior to speciation and have been conserved throughout evolution. *PhciCath2* and *5* cluster within a marsupial-specific clade, sister to that containing eutherian cathelicidins (Fig 2). Interestingly, *PhciCath5* is located in the clade containing *SahaCath3*, *5* and *6*, *ModoCath4*, and *MaeuCath1 and 7* (Fig 2), all of which display antimicrobial activity [30,35,56]. Focusing on the conserved cathelin domain, the inclusion of partial koala sequences *PhciCath7p* to *10p* does not influence the clustering of koala cathelicidins (S1 Fig). Although, *PhciCath5* now clusters with *PhciCath7p* to *10p* within the marsupial clade, forming a koala-specific expansion. The short branch lengths of *PhciCath5* and *PhciCath7p* to *10p* indicate that these genes likely arose through more recent duplications, compared to *PhciCath1*, *2*, *3* and *6* (S1 Fig). Although, *PhciCath7p* through *10p* may represent pseudogenes and hence not accurately portray phylogeny of functional koala cathelicidins.

**Fig 3:**
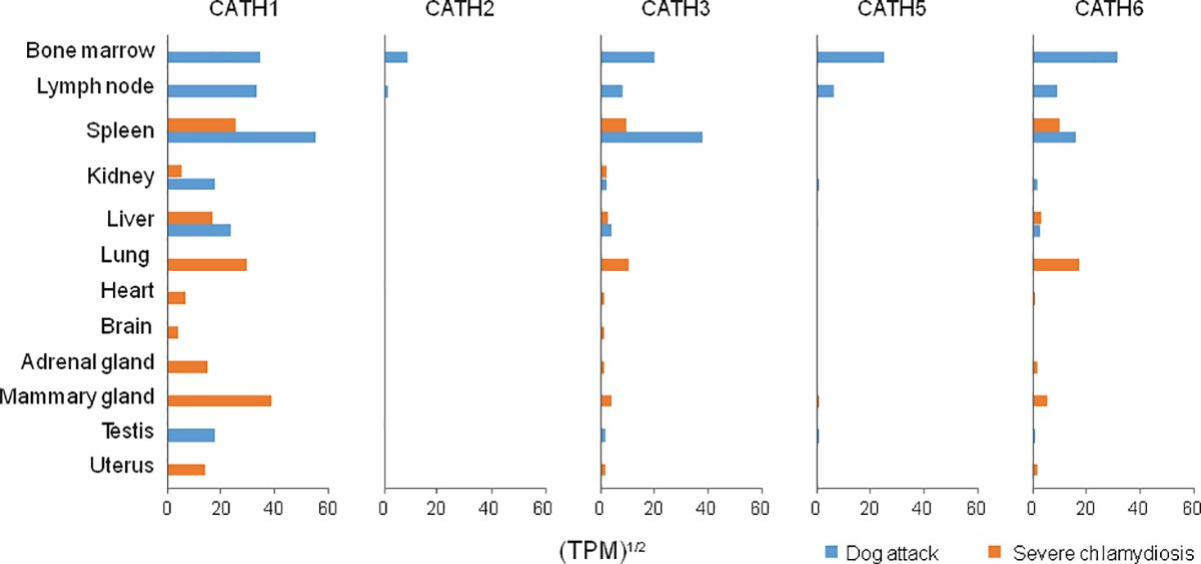
Expression of full-length koala cathelicidins in twelve tissue transcriptomes from two individuals [41,42]. Expressed as transcripts per million (TPM).

Only full-length cathelicidins *PhciCath1*, *2*, *3*, *5* and *6* were included in subsequent analyses as without the full coding sequence, partial sequences of *PhciCath7p* through *10p* could represent pseudogenes. Koala cathelicidins were transcribed in numerous tissues, similar to other marsupial [30,35] and eutherian cathelicidins [57]. Cathelicidin transcripts were detected in respiratory, cardiovascular, immune, reproductive and excretory tissues from two wild koalas (Fig 3) [42]. *PhciCath1* had the greatest expression of any cathelicidin and the greatest breadth, with transcripts present in all fifteen tissue transcriptomes (Fig 3). This broad expression of cathelicidins within multiple organ systems is likely derived from epithelial cells, which in humans constitutively express cathelicidins [57]. Here they likely provide rapid defence against infection, without the lag imposed by the recruitment and activation of neutrophils.

With the exception of *PhciCath1*, cathelicidin expression is favoured in immune tissues over non-immune tissues, although variation between the two individuals is marked (Fig 3). All five cathelicidins *PhciCath1*, *2*, *3*, *5* and *6* were expressed in the bone marrow, likely due to the presence of neutrophil precursors as observed in humans [58] and guinea pigs [59]. Expression of cathelicidins within neutrophils changes throughout cell development, and peaks during the myelocyte and metamyelocyte stage within the bone marrow [58,59]. Tammar wallaby *MaeuCath1* was also expressed in the bone marrow, and peak expression coincided with maturation of immune organs in pouch young [25]. All koala cathelicidins except *PhciCath2* were expressed in the lymph node, and a high number of *PhciCath3* transcripts were present in the spleen (Fig 3). A high level of cathelicidin expression within koala immune tissues is not surprising given their localised expression within neutrophils and epithelial cells, and similar results observed in other marsupials [25,30].

PhciCath1 and 6 proteins were present in the koala milk proteome, along with *PhciCath3* transcripts in the mammary gland [41], and hence may provide a direct source of immune compounds to developing young. Similar findings were reported by Morris *et al*. (2016) where cathelicidins were detected in a koala early lactation mammary gland transcriptome and late lactation milk proteome [41]. Cathelicidins were relatively abundant in late lactation, comprising 1.1% of peptides [41]. Tasmanian devil milk also contained cathelicidins [30], similarly tammar wallaby cathelicidins were expressed in the mammary gland throughout lactation [35]. The presence of cathelicidins within the milk of three marsupial species suggests this feature is conserved across different marsupial lineages, indicating these peptides may play an essential role in pouch young protection and development [25,30].

### Koala cathelicidin PhciCath5 shows direct antimicrobial activity

Koala cathelicidin PhciCath5 was the only peptide to display antimicrobial activity when screened against representative Gram negative and positive bacterial strains, with the most potent activity detected against *E*. *coli* (MIC 16μg/mL) and *S*. *aureus* (8μg/mL) isolates (Table 2). PhciCath5 also displayed antifungal activity against the ATCC strains *Candida parapsilosis* 22019 and *Candida krusei* 6258. The spectrum of activity was similar to that of other marsupial [30,35,60] and monotreme [35,60] cathelicidins. PhciCath5 was also active against the test strain of methicillin-resistant *Staphylococcus aureus* (MRSA) with an MIC of 16μg/mL. This MIC value is more potent than Tasmanian devil SahaCath5 against the same MRSA isolate [30], and within the range of MICs reported for human, bovine and rabbit cathelicidins against different MRSA isolates [61]. MRSA is a pathogen of major concern to human health [62], and antimicrobials such as cathelicidins provide novel alternatives for development as they generally do not induce resistance as observed with traditional antibiotics [61,63].

**Table 2:** Koala cathelicidin mature peptide PhciCath5 displays antimicrobial activity against bacteria and fungi from humans and animals, expressed as the minimum inhibitory concentration (MIC).

Strain	PhciCath5 MIC (μg/mL)
*P*. aeruginosa*	>64
*P*. *aeruginosa* ATCC27853	>64
*E*. coli*	16
*E*. *coli* ATCC25922	64 (11)
	22^a^
	>175^b^
*S*. aureus*	8
*S*. *aureus* ATCC29213	16 (11)
MRSA*	16
*S*. *pneumoniae* ATCC49619	>64
*S*. *pyogenes* ATCC19615	64
*S*. *agalactiae* ATCC12386	64
*S*. agalactiae*	64
*S*. *dysgalactiae*	>64
*S*. *lutetiensis*	>64
*S*. equi*	>64
*S*. *oralis*	>64
*S*. *salivarius*	64
*S*. *mutans*	>64
*L*. monocytogenes*	64
*P*. multocida*	>64
*K*. pneumoniae*	>64
*C*. *parapsilosis* ATCC 22019	32
*C*. *krusei* ATCC 6258	64
*C*. *glabrata*	>64
*C*. *albicans*	>64

The MIC of PhciCath1, 2, 3 and 6 was >64ug/mL for all bacteria and fungi tested.

*denotes animal isolate, otherwise human clinical isolates and ATCC strains were tested. MICs were obtained using MH II B that contains magnesium and calcium divalent cations. MICs in brackets were obtained using MHB without the additional of aforementioned divalent cations.

^a^ denotes MICs obtained using MHB with 10% foetal bovine serum.

^b^ denotes MICs obtained using MHB with 20% whole mouse blood.

Despite this promising activity profile *in vitro*, multiple inhibitors present within the *in vivo* environment are known to influence antimicrobial activity. Indeed, antibacterial activity of PhicCath5 against the *E*. *coli* ATCC strain was neutralised in 20% whole blood, resulting in an increase in the MIC from 64 μg/mL to >175μg/mL, and a reduction in the MIC in 10% FCS from 22μg/mL to 11μg/mL (Table 2). This indicates that PhciCath5 may bind non-specifically to proteins within the blood, sequestering the peptides, or is enzymatically degraded, both of which have been documented with human [64], rabbit and sheep cathelicidins [65].

As observed in eutherian cathelicidins [66,67], adherence of PhciCath5 to pathogens was facilitated by electrostatic interactions between positively charged cathelicidins and negatively charged head groups on the surface of bacterial cell membranes. Divalent cations bind to the negatively charged head groups, thereby preventing interaction with positively charged cathelicidins [68]. This is evidenced by a reduction in antimicrobial activity following the addition of magnesium and calcium divalent cations to the media. The MIC of PhciCath5 against *E*. *coli* increased five-fold in the presence of divalent cations, while the effect on the MIC against *S*. *aureus* was less pronounced (Table 2). Given that electrostatic interaction enables pathogen adherence, a high cationic charge often correlates with antimicrobial activity amongst many eutherian cathelicidins [69], however we found no such association amongst koala cathelicidins.

**Fig 4:**
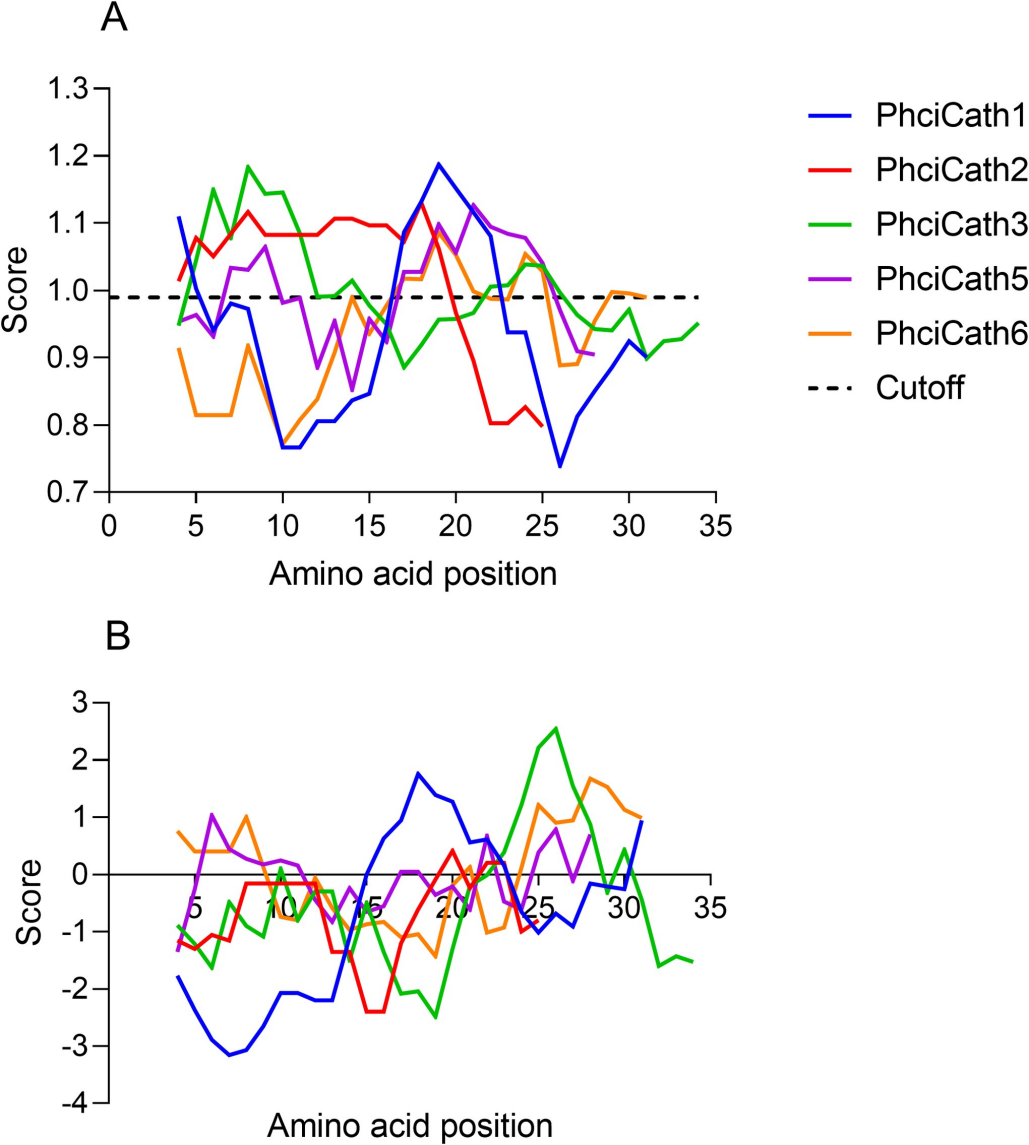
PhciCath5 contains two alpha helical regions according to the Deleage and Roux plot (A), as shown by the rise in scores above the 0.99 cutoff. PhciCath5 is also amphipathic according to the Kyte & Doolittle plot (B), with hydrophilic residues spanning the middle of the peptide (negative scores), and hydrophobic residues at the N- and C-terminus (positive scores). The remaining koala cathelicidin mature peptides all contain alpha helical regions (A), and vary in hydropathicity from largely hydrophilic (PhciCath2) to amphipathic (PhciCath1, 3, 5 and 6).

Following electrostatic attachment, PhciCath5 rapidly permeabilised bacterial cell membranes at high concentrations. At 44μg/mL, four times the MIC of 11μg/mL, PhciCath5 permeabilised ≥5% of the *E*. *coli* ATCC25922 cell membrane, leading to cell death within an hour of treatment (S2 Fig). However at the MIC, PhciCath5 was slow-acting, as the same level of membrane permeabilisation was only observed after three hours. This activity profile differs to tammar wallaby MaeuCath1 which rapidly killed bacteria at the MIC within 15 minutes [35]. The ability of eutherian cathelicidins to permeabilise bacterial cell membranes has been linked to an amphipathic alpha helical peptide structure [29,64,69]. The potent MaeuCath1 also forms an amphipathic alpha helix according to the predictive algorithms of Kyte and Doolittle, and Deleage and Roux [33,35]. Both algorithms suggest the same structure for PhciCath5 as observed in Fig 4A, with two alpha helical regions indicated by the scores rising above the 0.99 cutoff. While the negative GRAVY score suggests PhciCath5 is hydrophilic (Table 1), the Kyte and Doolittle hydropathicity plot reveals that PhciCath5 is amphipathic (Fig 4B). Hydrophilic residues span the middle of PhciCath5, with hydrophobic regions at the N and C-terminus. While PhciCath5 and MaeuCath1 both contain amphipathic alpha helical regions, additional physiochemical properties such as cationicity and sequence composition influence antimicrobial activity, and may explain the difference in activity and rate of permeabilisation between the two cathelicidins [69,70].

Permeabilisation of bacterial membranes by amphipathic alpha helical cathelicidins can be described by two models; the barrel stave model and the carpet model [66]. In the barrel stave model, aggregates of cathelicidins insert into the membrane and form transmembrane pores, thereby enabling leakage of essential molecules and disrupting transmembrane potential. Amphipathicity facilitates membrane insertion, as the hydrophobic surface of the peptide interacts with the lipid core of the bacterial cell membrane, and the hydrophilic surface forms the lining of the pore [66]. Alternatively, the carpet model does not involve peptide insertion. Instead cathelicidins bind to the surface of the membrane until a threshold concentration is reached, which disrupts the curvature of the membrane leading to destabilisation [66]. These results are speculative, and lipid membrane models would be required to confirm the mechanism of PhciCath5 membrane permeabilisation.

Koala cathelicidins PhciCath1, 2, 3 and 6 were inactive against all bacteria and fungi included in our assays at the concentrations tested. However, given the diversity and complexity of marsupial microbiomes known to contain novel and uncharacterised taxa [71–73], it is possible that they have activity against specific bacteria and fungi not tested in this study. Some marsupial cathelicidins have shown selective activity, such as Tasmanian devil SahaCath3 which was only active against *Cryptococcus neoformans* [30]. However, PhciCath1, 3 and 6 are orthologous to marsupial cathelicidins in which antimicrobial activity has not been demonstrated (Fig 2) [26,60]. Conservation of PhciCath1, 2, 3 and 6 suggests an essential function that has been conserved throughout marsupial evolution. The high level of expression in immune tissues (Fig 3) supports a role in modulating the immune response. While the immunomodulatory functions of marsupial cathelicidins remain to be tested, eutherian cathelicidins are chemotactic to various immune cells, modulate immune cell development and alter cytokine expression profiles [24].

### PhciCath5 is active against *Chlamydia pecorum*

**Fig 5:**
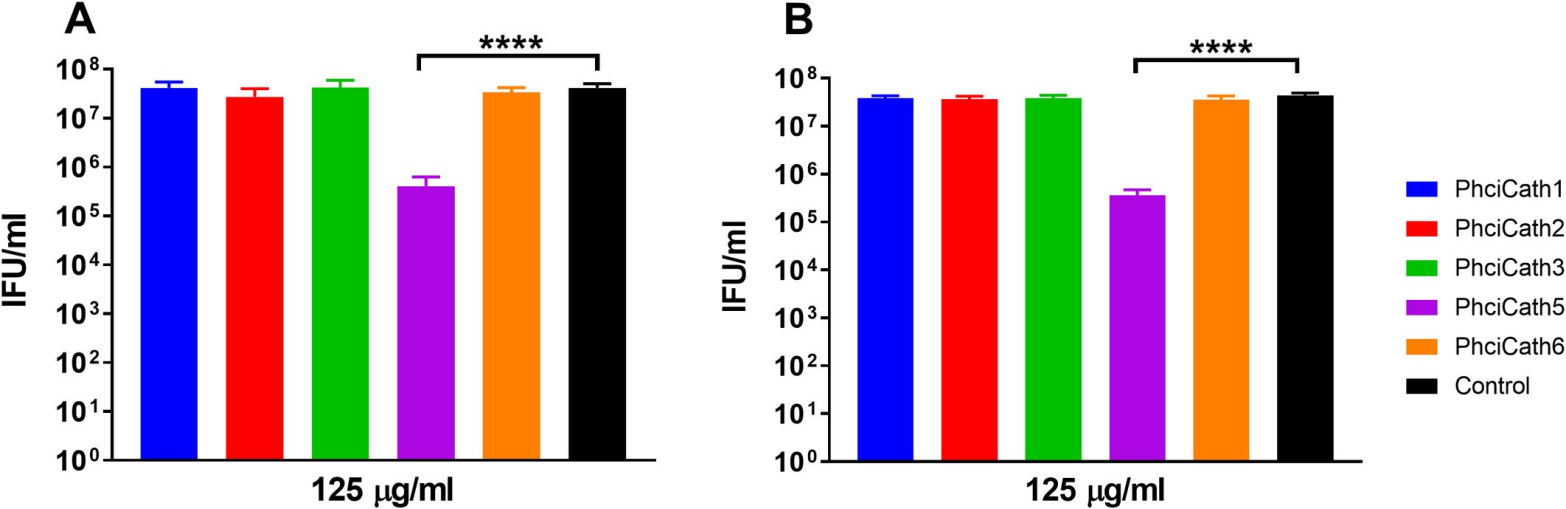
Activity of koala cathelicidins PhciCath1, 2, 3, 5 and 6 against *C*. *pecorum* MarsBar (A) and IPTaLE (B) at 125μg/mL. Expressed as inclusion forming units (IFU) per mL. Treatment of *C*. *pecorum* MarsBar and IPTaLE elementary bodies with 125μg/mL of PhciCath5 caused a significant decrease in infectious progeny in both serovars. PhciCath1, 2, 3 and 6 were ineffective against both serovars up to a concentration of 500ug/mL. **** indicates *p*<0.0001 significance was identified.

Koala cathelicidin PhciCath5 inactivated *C*. *pecorum* MarsBar and IPTaLE elementary bodies (EB) and was the only peptide tested that caused biologically and statistically significant reductions in chlamydial inclusions. Treatment with 125μg/mL of PhciCath5 resulted in a more than 2 orders of magnitude decrease in infectious progeny of both *C*. *pecorum* serovars, compared with the control (Fig 5). PhciCath1, 2, 3 and 6 were inactive at concentrations up to 500μg/mL, with less than half an order of magnitude difference in chlamydial inclusions compared with the control. Other marsupial cathelicidins have not been tested for anti-chlamydial activity and only a handful of studies have tested eutherian cathelicidins. They revealed wide variation in activity between chlamydial species and serovars [36,37,74,75]. Cathelicidins from humans and livestock inactivated a number of *C*. *trachomatis* isolates [36–39,74,75], especially pig protegrin PG-1 which reduced the infectivity of *C*. *trachomatis* at 1.25μg/mL [75]. Comparison of these results with koala cathelicidins presented in this study indicate the anti-chlamydial activity of PhciCath5 against *C*. *pecorum* is moderate at most, given PhciCath5 was active at over 100-fold higher concentration than PG-1, albeit against different *Chlamydia* species. However, experimental conditions used by Yasin et al 1996 differed from our study, as only a single round of infection was performed, and the reduction in inclusions or change in inclusion morphology measured. This is effectively a MIC, or the minimum cathelicidin concentration required to inhibit formation of chlamydial inclusions, but may not have killed the EB. In this study we conducted two rounds of infection, then measured the reduction in chlamydial infectivity. As such, our results effectively represent the minimum chlamydicidal concentration (MCC), or the minimum concentration of PhciCath5 which killed *Chlamydia* and hence reduced chlamydial infectivity [74]. Furthermore, the same eutherian cathelicidins which have activity against *C*. *trachomatis* were ineffective against one *C*. *pecorum* isolate at a maximum concentration of 80μg/mL [37]. However, eutherian cathelicidins have not been extensively tested against this *Chlamydia* species. Despite this, it is possible that koala cathelicidins evolved anti-chlamydial activity in response to host-pathogen co-evolution, and form part of the rapid innate defence at the mucosal surface.

Koala PhciCath5 acted directly upon, and inactivated *C*. *pecorum* MarsBar and IPTaLE EB. Removal of cathelicidins through centrifugation prior to chlamydial infection of the cell monolayer suggests PhciCath5 most likely induces permanent damage to the EBs within 2 hours, rather than preventing EB uptake into the host cell. Similar results were observed for pig protegrin-1 (PG-1) against three *C*. *trachomatis* serovars after a single round of infection [75]. This study revealed that PG-1 interacted directly with EBs and caused significant morphological changes, including membrane damage, loss of cytoplasm and nucleus [75]. Given that *Chlamydia* are Gram-negative bacteria, PhciCath5 may affect membrane permeability as it did in *E*. *coli*. However, *Chlamydia* EB have a strong, cross-linked outer membrane which differs substantially from the outer membrane of *E*. *coli* [75]. Because of its small size, only 31 residues in length, PhciCath5 may be able to penetrate through these structures and bind to the outer membrane of *C*. *pecorum*.

If PhciCath5 inactivates EBs, why are koalas with chlamydiosis unable to clear *C*. *pecorum* infection naturally? PhciCath5 and other koala cathelicidins may be present at the site of *Chlamydia* infection, secreted from epithelial cells or infiltrating immune cells. This is evidenced by expression in immune tissues such as the bone marrow, lymph node and spleen (Fig 2). Cathelicidins are expressed within neutrophils, lymphocytes and macrophages [76], all of which infiltrate the submucosa of the conjunctiva, urogenital and reproductive tract of the koala during infection [77]. However, it is unlikely that PhciCath5 reaches the effective concentration of 125μg/mL *in vivo* which inactivated EBs *in vitro*. The human cathelicidin LL-37 is present in plasma at a concentration of 1.2μg/mL [78] and bronchioalveolar lavage fluid up to 15μg/mL [79]. As such, cathelicidin expression at the site of *Chlamydia* infection may not be adequate to influence the progression of infection. Further work is required to quantify cathelicidin concentration at the site of infection in order to determine susceptibility *in vitro* at a representative concentration.

Our results show PhciCath5 has activity against extracellular EB. Timing of cathelicidin release from immune and epithelial cells within the host may not enable direct interaction with EB. Intracellular *Chlamydia* may be more resistant to cathelicidin attack, as treatment of intracellular *C*. *trachomatis* with PG-1 resulted in a 67% reduction in infectivity, compared to almost 100% reduction following treatment of extracellular EBs [74]. Indeed, proteases secreted by this *C*. *trachomatis* neutralise LL-37 anti-chlamydial activity, thereby evading AMP attack and ensuring extracellular EB survival. *Chlamydia* protease-like factor (CPAF) [38] and *Chlamydia* high temperature requirement protein A (cHtrA) [39] both specifically degrade LL-37. Whereas the plasmid encoded virulence factor pgp3 binds to, and forms stable complexes with LL-37, neutralising anti-chlamydial activity [80]. Interestingly, pgp3 also blocks LL-37 pro-inflammatory functions, which delays the inflammatory response and promotes *Chlamydia* survival [81]. Pgp3 also uses LL-37 to enhance its own pro-inflammatory activity on neutrophils, which may aid *Chlamydia* spreading [81]. CPAF, cHtrA and pgp3 are secreted into the cytoplasm of infected host cells and released upon host cell lysis, degrading or neutralising extracellular LL-37 before exposure of intra-inclusion EB [38,39,81]. *C*. *pecorum* also contains the genes which encode the aforementioned virulence factors [11,52,82,83], and both MarsBar and IpTaLE serovars possess the plasmid which encodes pgp3 [82,83]. While AMP evasion strategies have not been investigated in *C*. pecorum, given the presence of virulence factors and results in *C*. *trachomatis*, there is potential for the anti-chlamydial activity of PhciCath5 to be inactivated *in vivo*.

### Drug development potential

The broad-spectrum activity of PhciCath5 against bacteria and fungi, including drug-resistant MRSA, as well as *C*. *pecorum* suggests that it shows promise for development as a therapeutic. Peptide modification is required to identify residues responsible for antimicrobial activity, and those involved in non-specific binding to blood proteins, similar to the alanine scans performed for LL-37 and derivatives [84,85]. Additional assays are required to assess mammalian cell toxicity, one of the main barriers to cationic peptide development. A number of marsupial cathelicidins are cytotoxic, although mainly at concentrations far above the MIC [26,60]. Many eutherian cathelicidins are currently under pharmaceutical development as topical agents because they were associated with toxicity, low tissue penetration and peptide degradation when trialled for systemic use [86]. Derivatives of LL-37 and bovine indolicidin are currently in development as topical agents, while a topical formulation of PG-1 derivative known as Iseganan has reached phase III clinical trials for the treatment of oral mucositis [86]. Topical antibiotics are commonly used for the treatment of ocular chlamydiosis in koalas due to ease of application [87], hence topical cathelicidin formulations may provide alternative treatment options in the future.

Synergy between cathelicidins and traditional antibiotics has resulted in increased antimicrobial activity [24]. Perhaps the same is true for PhciCath5 and chloramphenicol, which is commonly used to treat chlamydiosis in koalas [21]. Given its broad-spectrum activity, topical application of PhciCath5 may also prevent or reduce secondary infections involving Gram-negative and Gram-positive bacteria or fungi, which have been reported in koala chlamydiosis [87].

## Conclusions

We characterised ten cathelicidins in the koala, five of which were full-length sequences that were widely expressed in tissues throughout the body. One cathelicidin, PhciCath5 displayed broad-spectrum antimicrobial activity against representative bacteria and fungi, including drug resistant strains. The activity of the remaining four cathelicidins may be highly specific or immunomodulatory. When tested against *Chlamydia*, PhciCath5 significantly reduced the infectivity of *C*. *pecorum* IPTaLE and MarsBar by inactivating elementary bodies prior to infection. Despite this, PhciCath5 may be unable to prevent or control *C*. *pecorum* infections in koalas due to inadequate peptide concentration at the site of infection, timing of peptide release or production of AMP-degrading proteases by *Chlamydia*. PhciCath5 represents a lead for antimicrobial development, with additional work required to confirm the absence of toxicity, explore potential synergistic effects with current antibiotics, and introduce peptide modifications that enhance antimicrobial activity.

## Supporting information

S1 Fig Koala cathelicidins cluster with other marsupials in the phylogenetic tree. The koala-specific expansion containing PhciCath5, and 7p to 10p, clusters with other marsupial cathelicidins that display antimicrobial activity. Sequences are coloured according to antimicrobial activity against bacteria and/or fungi; blue indicates active, red indicates inactive, black indicates peptide has not been tested. Only bootstrap values greater than 70% are shown. Accession numbers for sequences used are available in S3 Table.
(TIF)

S2 Fig Membrane permeabilization of PhciCath5 against *E*. *coli* ATCC25922. The concentration of PhciCath5 which resulted in ≥5% membrane permeability, relative to the positive control (lysis buffer), was reported. PhciCath5 permeabilized the *E*. *coli* cell membrane within an hour of treatment at 4x the MIC. The ≥5% permeability threshold was only reached after 3hrs at the MIC. Only values up to 100% permeability are shown, as at 4hrs post-treatment, membrane permeability at 2x the MIC reached 130% and 4x the MIC 316% relative to the positive control.
(TIF)

S1 Table Amino acid similarity amongst koala cathelicidin mature peptide sequences. (DOCX)

S2 Table Genomic coordinates of koala cathelicidin sequences. (DOCX)

S3 Table Sequence accession numbers used in BLAST searches and phylogenetic trees. See Figs 3 and S1.
(DOCX)
