# Supplementary figures and images for: Koala cathelicidin PhciCath5 has antimicrobial activity, including against Chlamydia pecorum
Source: PLoS One. 2021 Apr 14;16(4):e0249658. doi: 10.1371/journal.pone.0249658 (PMC8046226; doi:10.1371/journal.pone.0249658)

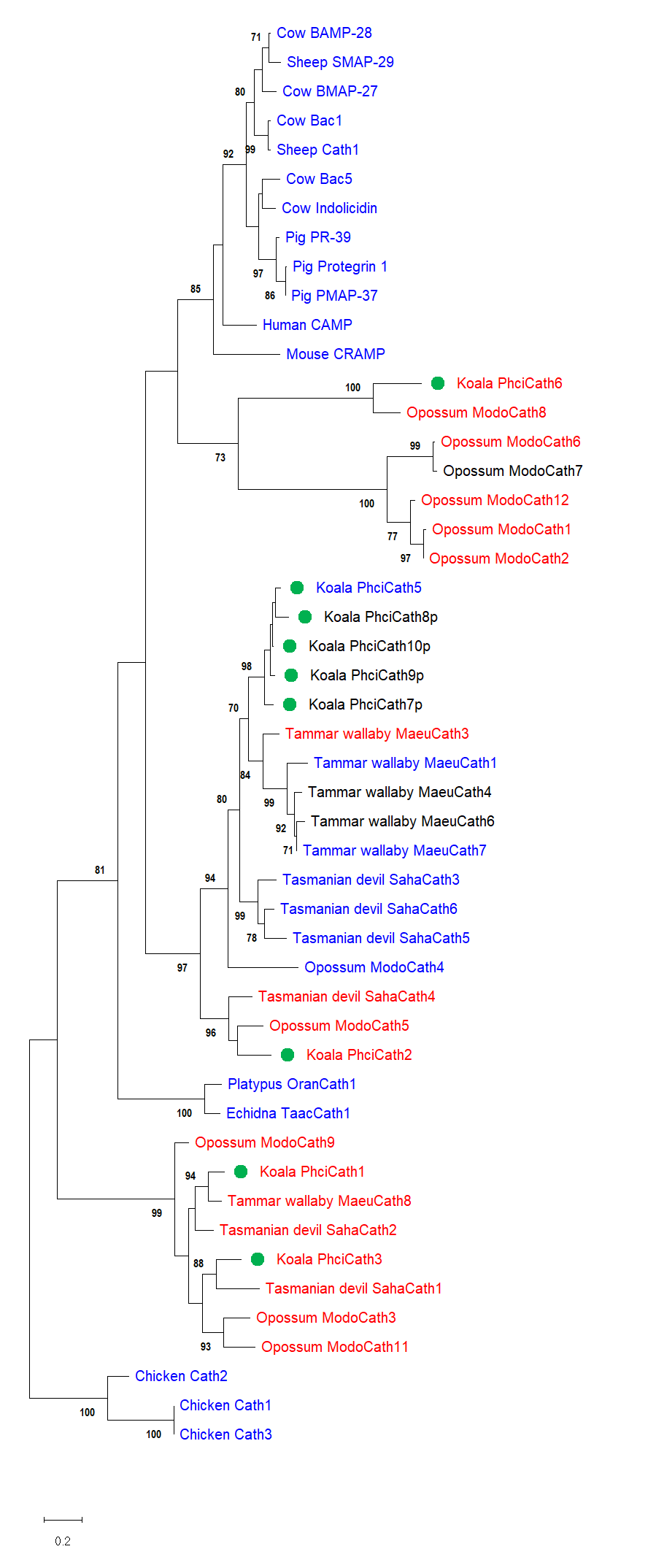

Supplement: S1 Fig — The koala-specific expansion containing PhciCath5, and 7p to 10p, clusters with other marsupial cathelicidins that display antimicrobial activity. Sequences are coloured according to antimicrobial activity against bacteria and/or fungi; blue indicates active, red indicates inactive, black indicates peptide has not been tested. Only bootstrap values greater than 70% are shown. Accession numbers for sequences used are available in S3 Table. (TIF) [file pone.0249658.s001.tif]

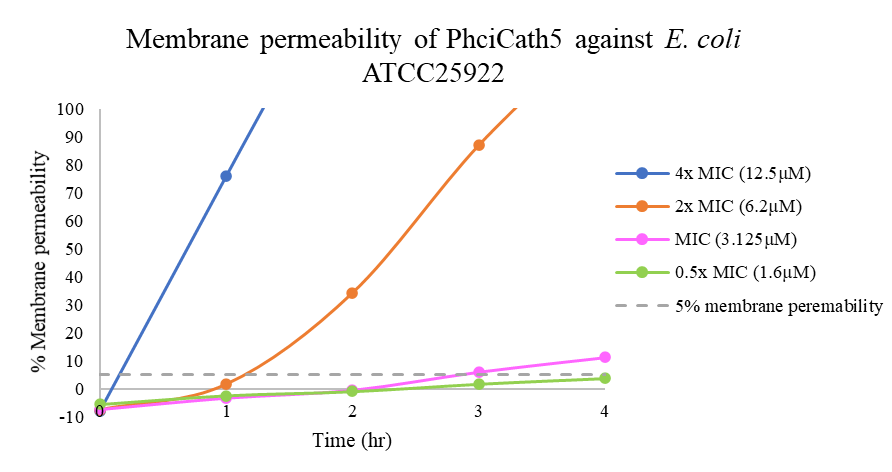

Supplement: S2 Fig — The concentration of PhciCath5 which resulted in ≥5% membrane permeability, relative to the positive control (lysis buffer), was reported. PhciCath5 permeabilized the E. coli cell membrane within an hour of treatment at 4x the MIC. The ≥5% permeability threshold was only reached after 3hrs at the MIC. Only values up to 100% permeability are shown, as at 4hrs post-treatment, membrane permeability at 2x the MIC reached 130% and 4x the MIC 316% relative to the positive control. (TIF) [file pone.0249658.s002.tif]
