# Supplementary material for: Koala cathelicidin PhciCath5 has antimicrobial activity, including against Chlamydia pecorum
Source: PLoS One. 2021 Apr 14;16(4):e0249658. doi: 10.1371/journal.pone.0249658 (PMC8046226; doi:10.1371/journal.pone.0249658)
Supplement: S1 Table — (DOCX) [file pone.0249658.s003.docx]

**S1 Table.** **Amino acid similarity amongst koala cathelicidin mature peptide sequences.**

|  | **PhciCath1** | **PhciCath2** | **PhciCath3** | **PhciCath5** | **PhciCath6** |
| --- | --- | --- | --- | --- | --- |
| **PhciCath1** | - | 11.7 | 30.0 | 5.8 | 19.4 |
| **PhciCath2** | 11.7 | - | 7.6 | 16.1 | 17.6 |
| **PhciCath3** | 30.0 | 7.6 | - | 17.9 | 15.3 |
| **PhciCath5** | 5.8 | 16.1 | 17.9 | - | 20.5 |
| **PhciCath6** | 19.4 | 17.6 | 15.3 | 20.5 | - |
