# Supplementary material for: Koala cathelicidin PhciCath5 has antimicrobial activity, including against Chlamydia pecorum
Source: PLoS One. 2021 Apr 14;16(4):e0249658. doi: 10.1371/journal.pone.0249658 (PMC8046226; doi:10.1371/journal.pone.0249658)
Supplement: S2 Table — (DOCX) [file pone.0249658.s004.docx]

**S2 Table**. **Genomic coordinates of koala cathelicidin sequences corresponding to the koala genome phaCin_unsw_v4.1.fa which is available here** [**https://asia.ensembl.org/Phascolarctos_cinereus/Info/Index**](https://asia.ensembl.org/Phascolarctos_cinereus/Info/Index)

| **Gene name** | **Start** | **End** | **Orientation** |
| --- | --- | --- | --- |
| PhciCath1 | 3071718 | 3071903 | + |
|  | 3073915 | 3074058 | + |
|  | 3074568 | 3074657 | + |
|  | 3075205 | 3075339 | + |
| PhciCath2 | 1695957 | 1695769 | - |
|  | 1695021 | 1694902 | - |
|  | 1694655 | 1694572 | - |
|  | 1693341 | 1693204 | - |
| PhciCath3 | 3086656 | 3086471 | - |
|  | 3085607 | 3085503 | - |
|  | 3084658 | 3084575 | - |
|  | 3084049 | 3083936 | - |
| PhciCath4-pseudogene | 3009356 | 3009168 | - |
|  | 3007757 | 3007656 | - |
|  | 3004230 | 3004143 | - |
| PhciCath5 | 1783748 | 1783560 | - |
|  | 1782060 | 1781926 | - |
|  | 1781673 | 1781602 | - |
|  | 1780599 | 1780495 | - |
| PhciCath6 | 3029447 | 3029621 | + |
|  | 3032102 | 3032204 | + |
|  | 3033684 | 3033774 | + |
|  | 3034799 | 3034918 | + |
| PhciCath7p | 1715719 | 1715531 | - |
|  | 1714038 | 1713925 | - |
|  | 1713670 | 1713598 | - |
| PhciCath8p | 1737360 | 1737172 | - |
|  | 1735651 | 1735523 | - |
|  | 1735292 | 1735220 | - |
| PhciCath9p | 1803700 | 1803512 | - |
|  | 1801746 | 1801627 | - |
| PhciCath10p | 1836969 | 1836790 | - |
|  | 1835300 | 1835181 | - |
|  | 1834930 | 1834861 | - |
