# Supplementary material for: Koala cathelicidin PhciCath5 has antimicrobial activity, including against Chlamydia pecorum
Source: PLoS One. 2021 Apr 14;16(4):e0249658. doi: 10.1371/journal.pone.0249658 (PMC8046226; doi:10.1371/journal.pone.0249658)
Supplement: S3 Table — See Figs 3 and S1. (DOCX) [file pone.0249658.s005.docx]

**S3 Table. Sequence accession numbers used in BLAST searches and phylogenetic trees.** See Fig. 3 and S2 Fig.

| **Species** | **Gene name** | **Accession number (ensembl, genbank, uniprot or publication reference)** |
| --- | --- | --- |
| Tasmanian devil  (*Sarcophilus harrisii*) | SahaCath1 | [1] |
|  | SahaCath2 |  |
|  | SahaCath3 |  |
|  | SahaCath4 |  |
|  | SahaCath5 |  |
|  | SahaCath6 |  |
| Tammar wallaby  (*Macropus eugenii*) | MaeuCath1 | EF624481.1 |
|  | MaeuCath3 | EF624483.1 |
|  | MaeuCath6 | EF624486 |
|  | MaeuCath7 | EF624487 |
|  | MaeuCath8 | [2] |
| Gray short-tailed opossum  (*Monodelphis domestica*) | ModoCath1 | [3] |
|  | ModoCath2 |  |
|  | ModoCath3 |  |
|  | ModoCath4 |  |
|  | ModoCath5 |  |
|  | ModoCath6 |  |
|  | ModoCath7 |  |
|  | ModoCath8 |  |
|  | ModoCath9 |  |
|  | ModoCath11 |  |
|  | ModoCath12 |  |
| Platypus  (*Ornithorhynchus anatinus*) | OranCath1 | [4] |
|  | OranCath2 |  |
| Echinda (*Tachyglossus aculeatus*) | TaacCath1 | [5] |
| Human (*Homo sapiens*) | CAMP | NM_004345.4 |
| Mouse (*Mus musculus*) | CRAMP | NM_009921.2 |
| Pig (*Sus scrofa*) | PMAP-37 | NM_001123149.1 |
|  | PR-39 | NM_214450.1 |
|  | Protegrin-1 | NM_001123149.1 |
| Sheep (*Ovis aries*) | Cath1 | NM_001009772.1 |
|  | SMAP-29 | L46854.1 |
| Chicken (*Gallus gallus*) | Cath1 | HQ640431.1 |
|  | Cath2 | AY817057.1 |
|  | Cath3 | HQ640433.1 |

# References

1. Peel E, Cheng Y, Djordjevic JT, Fox S, Sorrell TC, Belov K. Cathelicidins in the Tasmanian devil (*Sarcophilus harrisii*). Scientific Reports. 2016;6:35019.

2. Carman RL, Old JM, Baker M, Jacques NA, Deane EM. Identification and expression of an novel marsupial cathelicidins from the tammar wallaby (*Macropus eugenii*). Veterinary Immunology and Immunopathology. 2009;127:269-76.

3. Wong ESW, Papenfuss AT, Belov K. Immunome database for marsupials and monotremes. BMC Immunology. 2011;12(48):1-6.

4. Warren WC, Hillier LW, Graves JA, Binney E, Ponting CP, Grutzner F, et al. Genome analysis of the platypus reveals unique signatures of evolution. Nature. 2008;453:175-86.

5. Peel E, Cheng Y, Djordjevic JT, Kuhn M, Sorrell T, Belov K. Marsupial and monotreme cathelicidins display antimicrobial activity, including against methicillin-resistant *Staphylococcus aureus*. Microbiology. 2017;163:1457-65.
